# 48,XXYY syndrome presenting with long-term infertility and newly observed neck deformities: a case report

**DOI:** 10.1186/s13256-020-02375-z

**Published:** 2020-05-11

**Authors:** Mohammad Marwan Alhalabi, Marwan Alhalabi

**Affiliations:** 1 Department of Internal Medicine, Damascus University Faculty of Medicine, Fayez Mansour St., Damascus, Syria; 2 Department of Reproductive Medicine, Genetics and Embryology, Damascus University Faculty of Medicine, Fayez Mansour St., Damascus, Syria

**Keywords:** aneuploidy, sex chromosomes, clinical manifestations, diagnosis, genetics, infertility

## Abstract

**Background:**

Long-term infertility can be attributed to many factors, with the genetic factor being the most overlooked due to its many nonspecific morphological or endocrine signs. We present a rare case of a patient with progressive testicular failure associated with 48,XXYY syndrome.

**Case presentation:**

A 39-year-old Arab man presented to our fertility clinic for fertility treatment. He was diagnosed with primary infertility, which had been present for 20 years at the time of presentation. Our patient had nonspecific morphological features of an abnormally wide neck with front slouching neck posture, clinodactyly of the third finger, and had moderate hypoandrogenemic features. A semen analysis revealed azoospermia. Genetic tests for azoospermia, including sex-determining region Y (*SRY*) detection and chromosome Y microdeletion, revealed no deletion present on the Y chromosome. Karyotyping was used and our patient was diagnosed with 48,XXYY syndrome.

**Conclusion:**

Genetic testing (karyotyping and so on) played a key role in the diagnosis of our patient with long-term primary infertility secondary to 48,XXYY syndrome, and should play a vital role in all cases of long-term infertility, especially when presentation is accompanied by endocrine, skeletal, or morphological symptoms, signifying an underlying genetic factor.

## Background

48,XXYY syndrome was first described in the medical literature in 1960 by Muldal *et al.* [1] as an aneuploidy and a type of Klinefelter syndrome (47,XXY). Although phenotypically similar to Klinefelter syndrome, sharing features like hypergonadotropic hypogonadism [2], it is made distinct by symptoms of mental retardation and psychiatric disorders [3, 4]. The syndrome manifests later on in life with abdominal adiposity, small testicles, delayed development, behavioral disorders, learning disabilities, and delayed puberty, with such symptoms not presenting until early puberty [5–7]. Skeletal deformities were reported for 48,XXYY syndrome and could include radio-ulnar synostosis, osteoporosis, hyperostosis, pseudoepiphysis, kyphoscoliosis, and frontalis interna [8].

## Case presentation

**Fig. 1:**
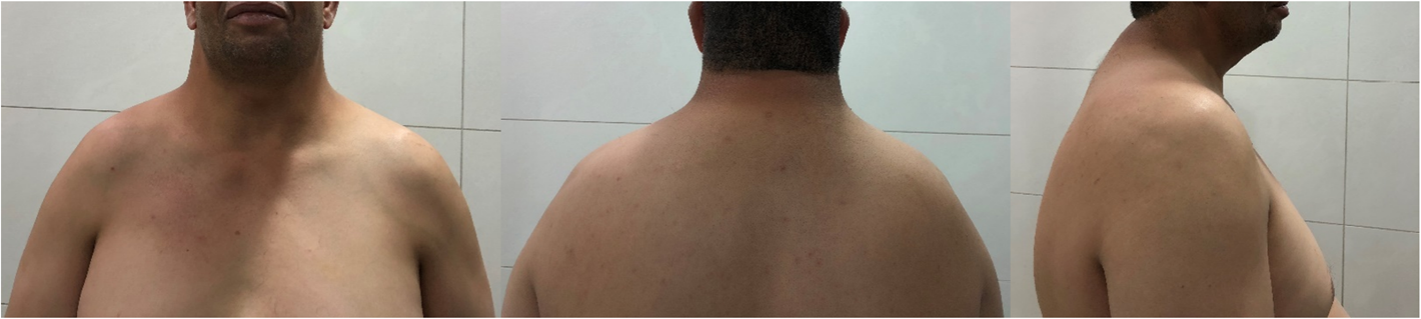
Our patient’s abnormal (webbed) neck shown in anterior, posterior, and lateral views

**Fig. 2:**
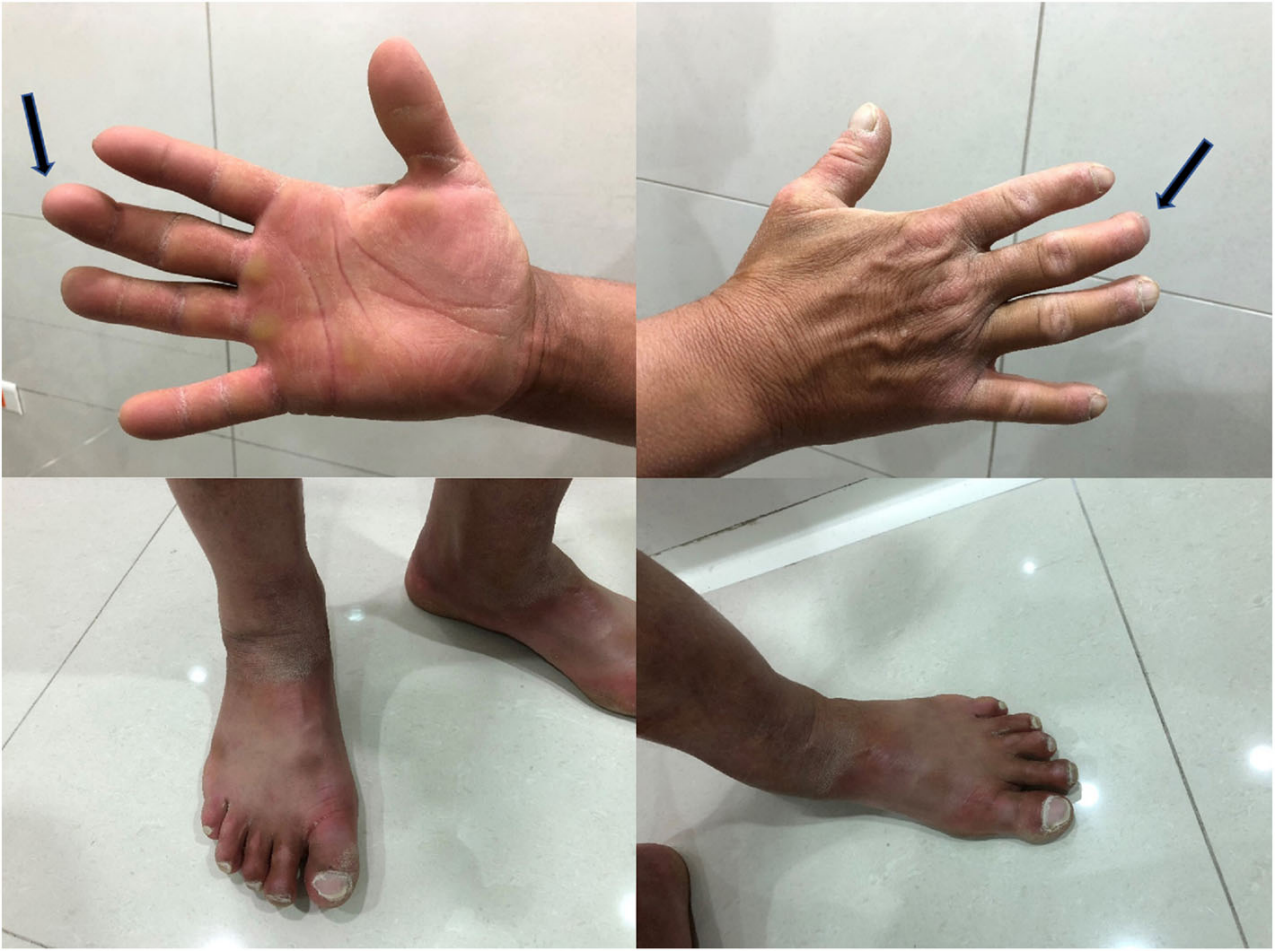
Our patient’s hands showing the clinodactyly of the third finger (black arrows) and the accompanying finger clubbing

**Fig. 3:**
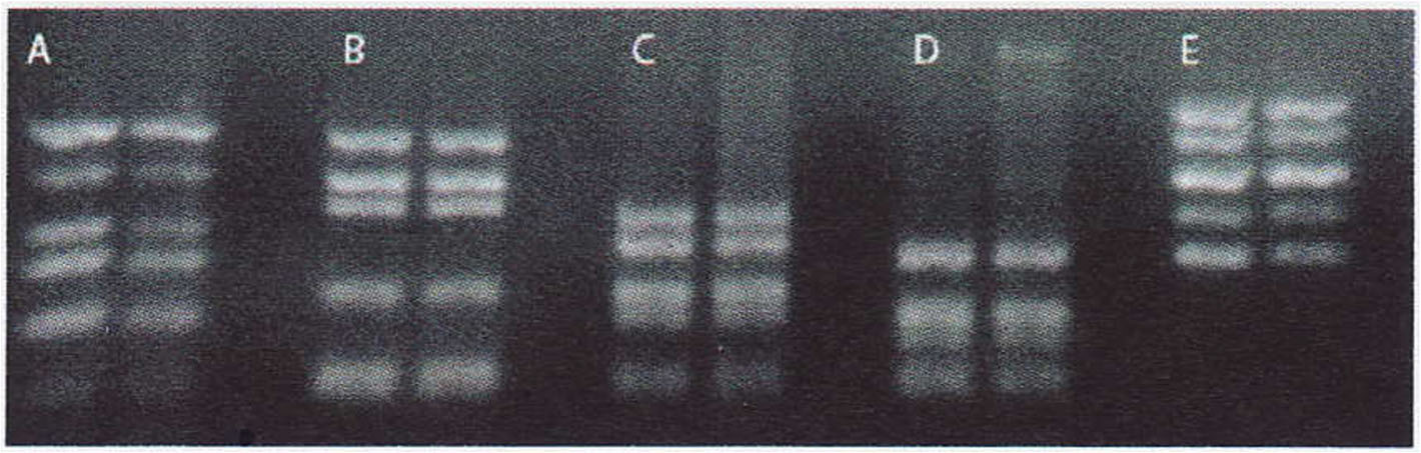
Molecular polymerase chain reaction (PCR) study showing no microdeletion in the Y chromosome

**Fig. 4:**
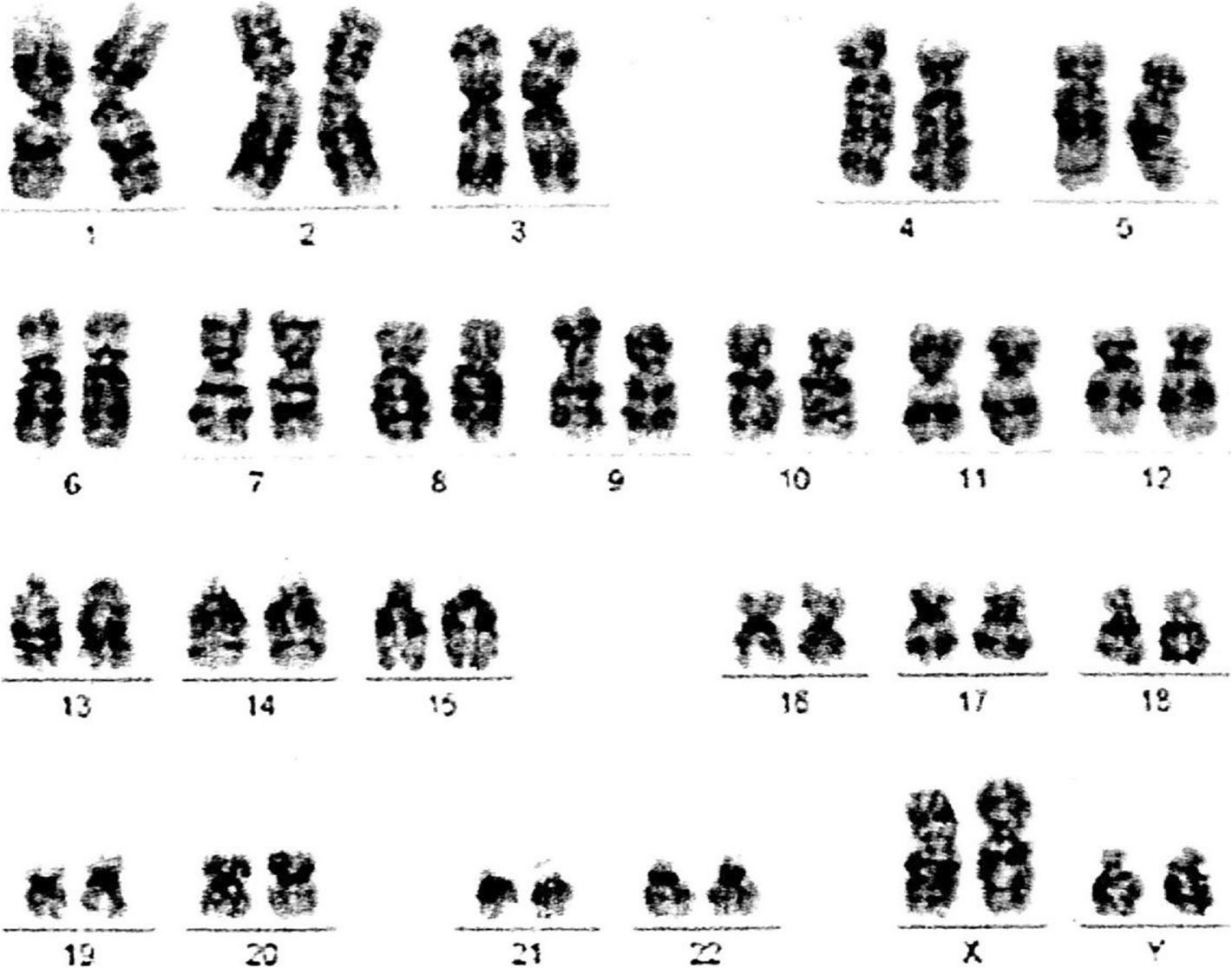
Peripheral blood karyotype of our patient showing 48,XXYY

**Fig. 5:**
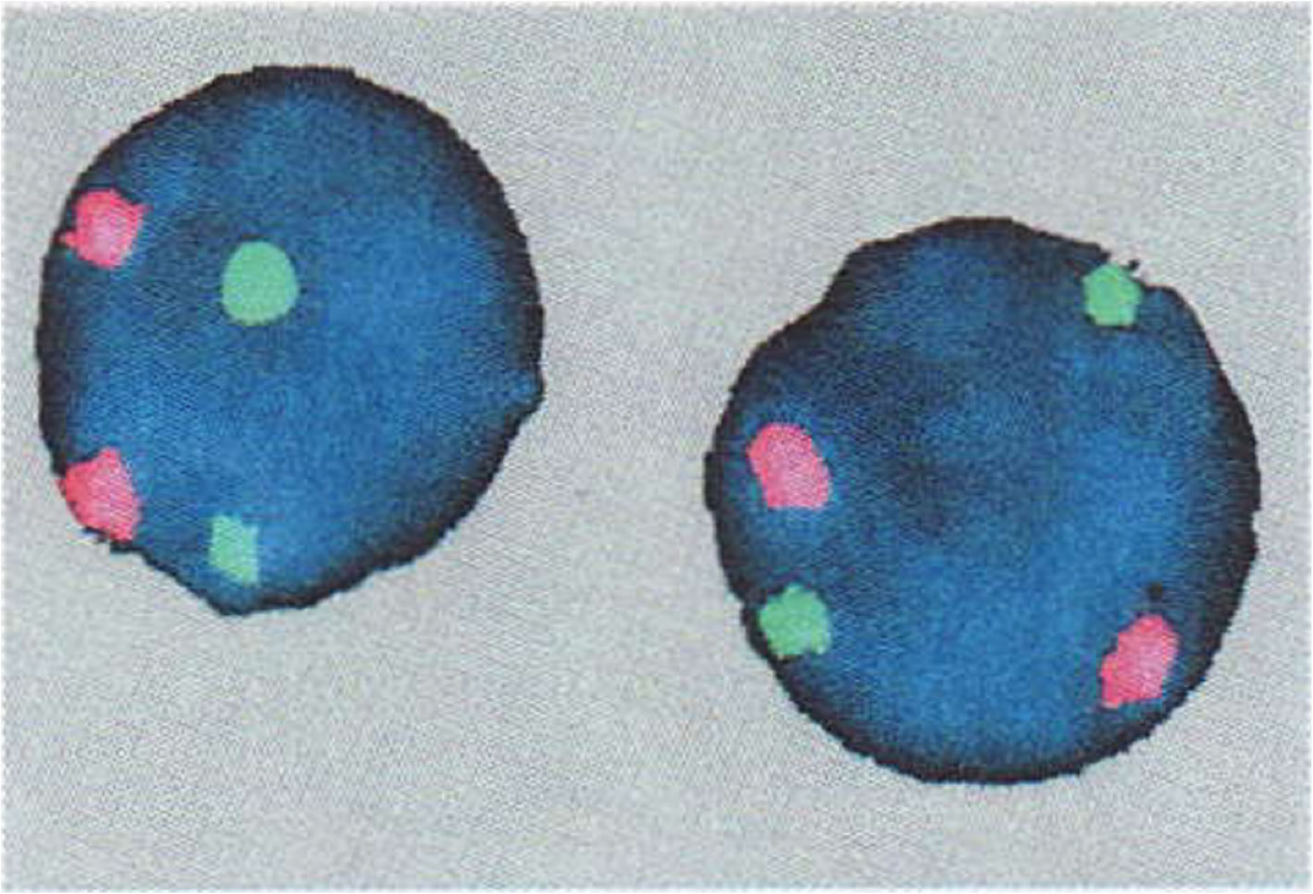
Fluorescence in situ hybridization (FISH) result showing the duplication of both X (*orange spectrum*) and Y (*green spectrum*) chromosomes

A 39-year-old Arab man presented to our fertility clinic for fertility treatment. Our patient was diagnosed with primary infertility, which had lasted for 20 years, and without successful conception in his marriage at the time of presentation. There was no history of pathologies, intervention, or medication that might have affected spermatogenesis. Upon physical examination our patient’s height was 160 cm and he weighed 85 kg (body mass index = 33.2), and he had an abnormally wide neck with a front slouching neck posture (Fig. 1). Our patient also had clinodactyly of the third finger and showed finger clubbing in all fingers and toes (Fig. 2). Our patient had moderate hypoandrogenemic features (slight facial and body hair), normal external genitalia, and small bilateral descended testes. Ultrasonography was conducted on our patient and revealed the volume of each testis was 4 ml without varicocele. Semen analysis revealed azoospermia, with blood testing showing normal complete blood count, and normal kidney and liver functions. The hormonal profile revealed low testosterone of 0.91 nmol/l (normal: 10.41–34.70 nmol/l), normal thyroid-stimulating hormone (TSH) of 1.9 mIU/l (normal: 0.4–4 mIU/l), normal prolactin levels of 11 ng/ml (normal: 2–18 ng/ml), elevated basal gonadotrophin levels [high follicular-stimulating hormone (FSH) of 30 IU/l (normal: 0–4 IU/l) and high luteinizing hormone (LH) of 19 IU/l (normal 1.24–7.8 IU/l)]; beta-human chorionic gonadotropin (hCG) detection was done, with a negative result. Microsurgical testicular sperm extraction (Micro-TESE) was conducted for sperm retrieval and did not find any mature spermatozoa. The pathological study of the biopsies found maturation arrest at the early stage of spermatogenesis. A molecular study to detect Y chromosome microdeletion with polymerase chain reaction (PCR) and gel electrophoresis was performed using the Y Chromosome Deletion Detection System Version 2.0 (Promega Corporation, Madison, WI, USA) and the result showed no deletion in any of the Y chromosomes (Fig. 3). Sex-determining region Y (SRY) detection was done by PCR, which revealed the presence of SRY in both Y chromosomes. The data and clinical features were suggestive of a sex chromosome aneuploidy. Karyotyping using high-resolution G-banding showed a case of aneuploidy with abnormality in the sex chromosomes, which was found to be 48,XXYY in all cells that were analyzed from the peripheral blood specimen (Fig. 4). Fluorescence in situ hybridization (FISH) to detect the X and Y chromosomes was done using a Vysis FISH Probe (Abbott Laboratories, Abbott Park, IL, USA) and revealed a duplication in both the X and Y chromosomes (Fig. 5). Although our patient had azoospermia, there was a strong requirement for conception from his family.

## Discussion

Despite XXYY syndrome initially being considered a variant of Klinefelter syndrome, now it is widely regarded as a separate clinical syndrome with psychological, morphological, and neurodevelopmental involvement [9–11]. While it is a sex chromosome aneuploidy, studies of live births reported a relatively rare incidence of 1:18000–1:50000 [3, 10]. Nonetheless, the case we present here is the first reported case in Syria and only two other cases have been reported in the region, in Turkey [12]. It was reported that the most prominent symptom in patients with Klinefelter syndrome was tall stature [13]. However, while tall stature was reported in 48,XXYY syndrome [7], it also concluded that patients with 48,XXYY syndrome were mostly of short stature, which is in accordance with our patient, who was not tall [2]. It was reported that patients with this syndrome suffered from infertility [14] due to its association with hypergonadotropic hypogonadism [3, 15] as well as its association with other endocrine manifestations such as acromegaloidism [16]. In our study, this concurred with our clinical findings, and our patient’s presenting symptom of infertility, which were suggestive for our patient to undertake genetic testing. It was also reported that the skeletal deformities that were present in 48,XXYY syndrome were the typical clinodactyly of the fifth finger [3], kyphoscoliosis of the spine [8], and one case reported an increased thickness of the neck [17]; but no other cases of 48,XXYY syndrome reported having an apparent abnormally wide neck characteristic of syndromes such as Noonan syndrome [18], front slouched neck posture, and clinodactyly of the third finger. To the best of our knowledge, this is the first case to report these features as accompanying symptoms for 48,XXYY syndrome.

## Conclusions

Our patient had a rare case of long-term primary infertility and progressive testicular failure secondary to 48,XXYY syndrome. Early diagnosis may have provided many treatment options before reaching the end stage of testicular failure. Genetic testing (karyotyping, and so on) plays a vital role in all cases of primary infertility presenting in the long term, especially when presentation is accompanied by endocrine, skeletal, morphological, or developmental symptoms, which could signify an underlying genetic factor.

## Data Availability

Data sharing not applicable to this article as no data sets were generated or analyzed during the current study.

## Abbreviations

FISH: Fluorescence in situ hybridization
Micro-TESE: Microsurgical testicular sperm extraction
PCR: Polymerase chain reaction
SRY: Sex-determining region Y
